# Association of immune inflammatory biomarkers with pathological complete response and clinical prognosis in young breast cancer patients undergoing neoadjuvant chemotherapy

**DOI:** 10.3389/fonc.2024.1349021

**Published:** 2024-02-06

**Authors:** Fucheng Li, Youyu Wang, He Dou, Xingyan Chen, Jianan Wang, Min Xiao

**Affiliations:** Department of Breast Surgery, Harbin Medical University Cancer Hospital, Harbin, China

**Keywords:** immune inﬂammatory biomarker, young women, breast cancer, pathological complete response, neoadjuvant chemotherapy, pan-immune-inﬂammation value (PIV)

## Abstract

**Background:**

The persistence of inflammatory stimulus has a tight relationship with the development of age-related diseases, ultimately resulting in a gradual escalation in the prevalence of tumors, but this phenomenon is rare in young cancer patients. Breast cancer arising in young women is characterized by larger tumor diameters and more aggressive subtypes, so neoadjuvant chemotherapy (NACT) can be especially appropriate for this population. Immune inflammatory biomarkers have been reportedly linked to the prognosis of some malignant tumor types, with varying results. In this study, we investigated the possible predictive value of blood-based markers in young breast cancer patients undergoing NACT, in addition to the association between the clinicopathological features and prognosis.

**Methods:**

From December 2011 to October 2018, a total of 215 young breast cancer patients referred to Harbin Medical University Cancer Hospital received NACT and surgery were registered in this retrospective study. The pretreatment complete blood counts were used to calculate the neutrophil-to-lymphocyte ratio (NLR), platelet-to-lymphocyte ratio (PLR), monocyte-to-lymphocyte ratio (MLR), and pan-immune-inflammation value (PIV).

**Results:**

NLR, PLR, MLR, and PIV optimal cut-off values were 1.55, 130.66, 0.24, and 243.19, as determined by receiver operating characteristic analysis. Multivariate analysis revealed that PIV, HR status, HER-2 status, and Ki-67 index were all independent predictive factors for pathological complete response. Subgroup analysis revealed that young breast cancer patients in the population characterized by low PIV and HR negative group were more likely to get pCR (P=0.001). The five-year overall survival (OS) rate was 87.9%, and Cox regression models identified PIV as independently related to OS.

**Conclusion:**

In the present study, the pretreatment PIV was found to be a useful prognostic indicator for pCR and long-term survival in young breast cancer patients undergoing NACT. High immune and inflammation levels, MLR and PIV were connected to poor clinical prognosis in young breast cancer patients. PIV is a promising biomarker to guide strategic decisions in treating young breast cancer.

## Highlights

- Immune inflammatory biomarkers are investigated solely in young patients with breast cancer.
- High immune and inflammation levels are connected to poor clinical prognosis in young breast cancer patients.
- The pretreatment pan-immune-inflammation value (PIV) is associated with pathological complete response and clinical prognosis.
- HR status, HER-2 status, and Ki-67 index are all independent predictive factors for pathological complete response in young breast cancer patients undergoing neoadjuvant chemotherapy.
- PIV is a promising biomarker to guide strategic decisions in treating young breast cancer.

## 1 Introduction

By 2020, breast cancer has overtaken lung cancer as the most common malignant tumor worldwide, with more than 600,000 deaths (1). Breast cancer in young females was defined as being diagnosed before the age of 40 (2), and about 16.4% of the females were diagnosed with young breast cancer in China, with a slight increase in the incidence over the past few years (3). Compared with elderly counterparts, young breast cancer patients have larger tumor diameters, more aggressive subtypes, and poorer biological behavior (4–6). Therefore, neoadjuvant chemotherapy (NACT) is especially suitable for this group of patients. Numerous NACT regimens have been used in the therapeutic management of breast cancer (7); nevertheless, no universally embraced international standard exists for evaluating the effectiveness of NACT in young patients with breast cancer.

The prognosis of breast cancer is closely related to some immunologic and histologic indicators. However, these biomarkers are arduous and expensive to obtain, commonly used in foundation research, greatly limiting their clinical application (8, 9). In contrast, peripheral blood counts are simpler to acquire and indicate systemic immune and inflammatory conditions. Immune inflammatory biomarkers (IIBs), encompassing the levels of neutrophil (N), platelet (P), monocyte (M), and lymphocyte (L), in conjunction with the neutrophil-to-lymphocyte ratio (NLR), platelet-to-lymphocyte ratio (PLR), and monocyte-to-lymphocyte ratio (MLR), have been emerged as prognostic factors for various malignant tumors (10–14). Pan-immune-inflammation value (PIV) is a new comprehensive biomarker calculated from neutrophil, monocyte, platelet, and lymphocyte counts (PIV=N × M ×P/L) that outperforms superior other separate indicators of immune-inflammatory in prognosticating clinical outcomes (15).

Older adults are often accompanied by dysfunction of the immune system, which is called immunosenescence (16). Immunosenescence is a typical physiological phenomenon linked to chronic low-grade inflammation (17, 18). During aging, organisms often showcase a distinct inflammatory state, characterized by a noteworthy expression of pro-inflammatory markers (17), which means lower levels of inflammation in healthy youngsters. At the same time, inflammation plays a beneficial role in removing harmful factors in early life and adulthood (19). However, studies related to immune and inflammation have not been widely investigated solely in young cancer patients.

Drawing upon previous investigations, we posit that IIBs could potentially assume a significant part in breast cancer prognosis. Consequently, we embarked upon this retrospective investigation to explore the prognostic role of IIBs exclusively in young breast cancer patients receiving NACT, to furnish a predictive and convenient indicator for pathological complete response (pCR) and survival outcomes.

## 2 Materials and methods

### 2.1 Patient selection and data collection

Before receiving treatment, each patient had duly endorsed the informed consent form regarding the subsequent utilization of their medical information or biological specimens. Furthermore, all activities involving human subjects within the study complied with the standards set forth by the committee, in strict accordance with the Helsinki Declaration of 1964 and other amendments to ethical standards.

As a retrospective study, we enrolled 215 young breast cancer patients, aged 40 or below when diagnosed, who received NACT and surgery in this trial at Harbin Medical University Cancer Hospital from December 2011 to October 2018. From each patient’s medical record, the specific treatment information, clinical data, and demographic information were retrieved. Patients’ peripheral blood was drawn from their veins within one week before NACT, and lymphocyte (L), monocyte (M), platelet (P), and neutrophil (N) counts were collected retrospectively to calculate NLR, PLR, MLR, and PIV.

The following were the patients’ inclusion criteria: 1) ≤ 40 years old; 2) pathological confirmation of invasive breast cancer diagnosis through core needle biopsy before undergoing NACT; 3) all patients received NACT and surgery in our hospital; 4) each patient’s clinical records and follow-up data were full.

The following were the patients’ exclusion criteria: 1) prior to receiving NACT, patients received chemotherapy, radiation, endocrine therapy, or targeted therapy; 2) patients with distant metastases; 3) patients with autoimmune or chronic inflammatory disease; 4) patients who started NACT within one month of receiving a blood transfusion.

### 2.2 Chemotherapy regimens

Following diagnosis, chemotherapy regimens were chosen for each patient based on immunohistochemical results, patient’s preferences, and financial situation. Our study took anthracycline- (A-) based and/or taxane- (T-) based NACT regimens, and a limited cohort of patients exhibiting human epidermal growth factor receptor-2 (HER-2) positivity received anti-HER-2 therapy. Repeat the cycle every three weeks for the selected regimens. All patients underwent a minimum of four cycles of NACT, followed by 2-3 weeks of respite before proceeding to surgery. It should be mentioned that only a portion of the patients received trastuzumab and pertuzumab due to financial difficulties. The recommended duration of treatment with anti-HER-2 therapy is one year, and completion of separate anti-HER-2 therapy should be continued for a full year after surgery. In this study, anti-HER-2 drugs were used concomitantly with paclitaxel-based chemotherapeutic agents, and anthracyclines were not used concomitantly with trastuzumab due to cardiotoxicity.

Antracycline and taxane regimens include: AC-T regimen: anthracyclines 100mg/m^2^, cyclophosphamide (C) 600mg/m^2^, followed by docetaxel 80-100mg/m^2^; TAC regimen: taxanes 75mg/m^2^, anthracyclines 50mg/m^2^, and cyclophosphamide 500mg/m^2^; AT regimen: taxanes 75mg/m^2^ and doxorubicin 60mg/m^2^.

Chemotherapy and trastuzumab regimens include: AC-TH regimen: anthracyclines 100mg/m^2^, cyclophosphamide 600mg/m^2^, followed by docetaxel 80-100mg/m^2^ and herceptin (H) 8mg/kg for the first time, then 6mg/kg; THP regimen: docetaxel 80-100mg/m^2^, herceptin 8 mg/kg for the first time, then 6 mg/kg and pertuzumab (P) first dose 840 mg, then 420 mg. TH regimen: docetaxel 80-100mg/m^2^, herceptin (H) 8 mg/kg for the first time, then 6 mg/kg.

Other regimens include: AC regimen: anthracyclines 90mg/m^2^ and cyclophosphamide 600mg/m^2^; TC regimen: docetaxel 80-100mg/m^2^ and cyclophosphamide 600mg/m^2^.

### 2.3 Classification criteria and response assessment

The eighth edition of the American Joint Committee on Cancer (AJCC) was used to assess the tumor clinical stage of each patient (20), which was the latest staging standard for breast cancer. The status of estrogen receptor positive (ER), progesterone receptor (PR), HER-2, Ki-67, and P53 were evaluated by immunohistochemistry (IHC) staining or *in situ* hybridization (ISH). Hormone receptor (HR) positive was defined as ER positive and/or PR positive. High expression of Ki-67, characterized by a nuclear positivity of >14%, was delineated, while low expression was classified as ≤14%, based on the St Gallen International Expert Consensus on the Primary Therapy of Early Breast Cancer 2013 (21). HER-2 status was divided into positive HER-2 and negative HER-2. IHC 0, IHC 1+, or IHC 2+ and ISH negative were defined as HER2 negative, and IHC 3+ or IHC 2+ and ISH positive were defined as HER-2 positive. In this study, the histological response was assessed using the Miller and Payne grade (MPG), and pCR was described as the absence of any residual invasive cancer cells within both the mammary and the lymph nodes (22).

### 2.4 Follow-up and endpoints

For the first two years post-surgery, outpatients checked on all patients every three months. Subsequently, follow-up appointments were scheduled biannually for the following three to five years, and then annual assessments were conducted every year until death. The deadline for this study’s follow-up was September 1, 2023. Patients who were lost to follow-up were excluded from this study, and 215 patients who met the enrollment criteria were ultimately included. Overall survival (OS) was defined as the date from the initial diagnosis to death or the end of follow-up.

### 2.5 Statistical analysis

All statistical processing and analyses were conducted using the SPSS software (version 26.0) and GraphPad prism software (version 8.0). Frequencies and percentages were used to describe categorical variables, and the median (interquartile range) was used to describe continuous variables. Receiver operating characteristic (ROC) analysis assessed the optimal cut-off value for IIBs. The optimal metric cutoff in the ROC curve makes for the best classification, with the optimal metric cutoff at the maximum value of the Yoden index. Univariate and multivariate analyses of the correlation between clinicopathological features and pCR were conducted by the logistic regression model. To avoid overfitting and underfitting, common important variables were included in the model. The associations between different PIV and HR subgroups were assessed by chi-square test. The cox proportional hazards regression model (univariate and multivariate) was used to examine the independent prognostic factors. OS associations were evaluated using the Kaplan–Meier methodology and log-rank test. The two-tailed *P*<0.05 was considered to be statistically significant.

## 3 Results

### 3.1 Young breast cancer patients’ clinicopathological characteristics

A total of 215 eligible young patients with breast cancer (≤40 years of age) were enrolled in this study. The median age was 36 years at diagnosis, and 76 cases (35.3%) were in the high body mass index (BMI) group (BMI>24). Most of the patients were clinical T2 stage (68.3%) and node positive (cN+, 84.7%) cancers, and the number of patients with clinical TNM (cTNM) III stage was 112 (52.0%), occupying more than half of the whole population. All of the young patients with HR positive, HER-2 negative, and Ki-67>14 was 147 (68.4%), 144 (67.0%), and 164 (76.3%), respectively. In a previously conducted prognostic analysis of breast cancer after NACT, T2 stage, cN+, and HER-2 negative accounted for 63.8%, 49.1%, and 74.2% in the overall population, respectively (23). There were 179 patients (83.2%) treated with antracycline and taxane regimen, and 24 patients (11.2%) received chemotherapy and trastuzumab regimen. About sixty percent of patients received more than or equal to six cycles of NACT (n = 131), and nearly sixteen percent underwent breast-conserving surgery (n = 34). At final histology after surgery, 51 (23.7%) patients obtained pCR (**Table 1**).

**Table 1:** Clinicopathological characteristics of young breast cancer patients.

Variable	N (n=215)	Variable	N (n=215)
**Age (median)**	36(23-40)	**Ki-67**	
**BMI**		≤14%	51(23.7%)
≤24	139(64.7%)	>14%	164(76.3%)
>24	76(35.3%)	**P53**	
**cT stage**		Negative	119(55.3%)
T1	25(11.6%)	Positive	96(44.7%)
T2	147(68.3%)	**Chemotherapy regimen**	
T3	34(15.8%)	Antracycline + Taxane	179(83.2%)
T4	9(4.1%)	Chemotherapy + Trastuzumab	24(11.2%)
**cN stage**		Other regimens	12(5.6%)
N0	33(15.3%)	**Chemotherapy Cycle**	
N1	82(38.1%)	<6	84(39.1%)
N2	65(30.2%)	≥6	131(60.9%)
N3	35(16.2%)	**Surgery**	
**cTNM stage**		Mastectomy	181(84.2%)
I	4(1.8%)	Conservative surgery	34(15.8%)
II	99(46.0%)	**pCR**	
III	112(52.0%)	No	164(76.3%)
**HR**		Yes	51(23.7%)
Negative	68(31.6%)	**NLR**	2.0(0.2-9.0)
Positive	147(68.4%)	**PLR**	134.2(51.5-401.9)
**HER-2**		**MLR**	0.2(0.1-0.7)
Negative	144(67.0%)	**PIV**	227.0(24.5-1681.5)
Positive	71(33.0%)		

### 3.2 Analysis of pCR by univariate and multivariate

The optimal cut-off values of IIBs for prediction of pCR were determined by receiver operating characteristic (ROC) analysis grouping by NLR (1.55), PLR (130.66), MLR (0.24), and PIV (243.19), with area under curve (AUC) values of 0.567, 0.608, 0.603, and 0.608, respectively (**Table 2**). From the ROC curve analysis, the AUC of PIV was the largest than NLR and MLR, indicating that PIV has a higher prognostic ability for pCR than NLR and MLR.

**Table 2:** Optimal cutoff values of NLR, PLR, MLR and PIV based on ROC curve analysis for prediction of pCR in patients.

Curve	Cut-off value	AUC	Sensitivity	Specificity	*P* value
NLR	1.55	0.567	0.333	0.805	0.149
PLR	130.66	0.608	0.647	0.579	**0.019**
MLR	0.24	0.603	0.765	0.451	**0.027**
PIV	243.19	0.608	0.725	0.512	**0.020**

Bold values indicate that they are statistically significant at P<0.05

Univariate analysis showed that all low IIBs subgroups were more likely to achieve pCR than the high IIBs subgroups (NLR: OR = 0.485, 95% CI 0.241-0.975, *P* value = 0.042; PLR: OR = 0.396, 95% CI 0.206-0.761, *P* value = 0.005; MLR: OR = 0.374, 95% CI 0.183-0.766, *P* value = 0.007; PIV: OR=0.360, 95% CI 0.181-0.716, *P* value = 0.004). Young breast cancer patients who were HR negative, HER-2 positive, and Ki-67>14% were more likely to reach pCR. In the multivariate analysis, indexes exhibiting statistically significant differences in the univariate analysis were included. Logistic regression analysis indicated that compared with the high PIV group, the low PIV group was more likely to obtain pCR (OR = 0.349, 95% CI 0.147-0.828, *P* value = 0.017), and this finding had statistical significance. The rate of pCR was significantly associated with the HR subgroup (OR = 0.214, 95% CI 0.102-0.453, *P* value <0.001), HER-2 subgroup (OR = 2.155, 95% CI 1.013-4.586, *P* value = 0.046), and Ki-67 subgroup (OR = 2.918, 95% CI 1.013-8.410, *P* value = 0.047). Our results revealed that PIV, HR status, HER-2 status, and Ki-67 index were independent predictors for pCR (**Table 3**).

**Table 3:** Univariate and multivariate analysis of clinical characteristics and IIBs in relation to pCR.

Variable	pCR(n=51)	Univariate analysis HR(95% CI)	*P* value	Multivariate analysis HR(95% CI)	*P* value
Age					
≤35	21(41.1%)	Ref.			
>35	30(58.8%)	1.175(0.621-2.221)	0.620		
BMI					
≤24	32(62.7%)	Ref.			
>24	19(37.2%)	1.115(0.581-2.140)	0.744		
cT stage					
T1+T2	44(86.2%)	Ref.			
T3+T4	7(13.7%)	0.566(0.235-1.363)	0.204		
cN stage					
N0	9(17.6%)	Ref.			
N+	42(82.3%)	0.800(0.345-1.853)	0.603		
cTNM stage					
I-II	27(52.9%)	Ref.			
III	24(47.0%)	0.768(0.409-1.441)	0.411		
HR					
Negative	29(56.8%)	Ref.		Ref.	
Positive	22(43.1%)	0.237(0.122-0.458)	**<0.001**	0.214(0.102-0.453)	**<0.001**
HER-2					
Negative	27(52.9%)	Ref.		Ref.	
Positive	24(47.0%)	2.213(1.160-4.220)	**0.016**	2.155(1.013-4.586)	**0.046**
Ki-67					
≤14%	5(9.8%)	Ref.		Ref.	
>14%	46(90.1%)	3.586(1.341-9.592)	**0.011**	2.918(1.013-8.410)	**0.047**
P53					
Negative	27(52.9%)	Ref.			
Positive	24(47.0%)	1.136(0.605-2.134)	0.692		
Chemotherapy regimen					
Antracycline + Taxane	40(78.4%)	Ref.			
Chemotherapy + Trastuzumab	9(17.6%)	2.085(0.849-5.119)	0.109		
Other regimens	2(3.9%)	0.655(0.146-3.302)	0.647		
Chemotherapy Cycle					
<6	17(33.3%)	Ref.			
≥6	34(66.6%)	1.381(0.714-2.673)	0.337		
Surgery					
Mastectomy	40(78.4%)	Ref.			
Conservative surgery	11(21.5%)	1.686(0.758-3.751)	0.201		
NLR					
≤1.55	17(33.3%)	Ref.		Ref.	
>1.55	34(66.6%)	0.485(0.241-0.975)	**0.042**	0.746(0.302-1.842)	0.526
PLR					
≤130.66	33(64.7%)	Ref.		Ref.	
>130.66	18(35.2%)	0.396(0.206-0.761)	**0.005**	0.629(0.283-1.396)	0.254
MLR					
≤0.24	39(76.4%)	Ref.		Ref.	
>0.24	12(23.5%)	0.374(0.183-0.766)	**0.007**	0.649(0.270-1.562)	0.335
PIV					
≤243.19	37(72.5%)	Ref.		Ref.	
>243.19	14(27.4%)	0.360(0.181-0.716)	**0.004**	0.349(0.147-0.828)	**0.017**

Bold values indicate that they are statistically significant at P<0.05

### 3.3 Association between different PIV groups and clinicopathological characteristics

With the above findings, we have already known that PIV was particularly associated with pCR. We further explored the relationship between different PIV groups and clinicopathological features. Low PIV value was significantly associated with pCR (χ^2 ^= 8.860, *P* = 0.003) and has no statistical significance with other clinicopathological characteristics. It is worth noting that the difference between PIV and HER-2 subgroups was close to reach statistical significance (χ^2 ^= 3.735, *P* = 0.053) (**Table 4**).

**Table 4:** Relationship between clinicopathological characteristics and different PIV groups.

Variable	N (n=215)	PIV ≤ 243.19 (n=117)	PIV>243.19 (n=98)	χ^2^	*P* value
**Age**				0.553	0.457
≤35	95(44.2%)	49(41.9%)	46(46.9%)		
>35	120(55.8%)	68(58.1%)	52(53.1%)		
**BMI**				2.356	0.125
≤24	139(64.7%)	81(69.2%)	58(59.2%)		
>24	76(35.3%)	36(30.8%)	40(40.8%)		
**cT stage**				0.675	0.411
T1+T2	172(80.0%)	96(82.1%)	76(77.6%)		
T3+T4	43(20.0%)	21(17.9%)	22(22.4%)		
**cN stage**				2.358	0.125
N0	33(15.3%)	22(18.8%)	11(11.2%)		
N+	182(84.7%)	95(81.2%)	87(88.8%)		
**cTNM stage**				1.172	0.279
I-II	103(47.9%)	60(51.3%)	43(43.9%)		
III	112(52.1%)	57(48.7%)	55(56.1%)		
**HR**				1.391	0.238
Negative	68(31.6%)	33(28.2%)	35(35.7%)		
Positive	147(68.4%)	84(71.8%)	63(64.3%)		
**HER-2**				3.735	0.053
Negative	144(67.0%)	85(72.6%)	59(60.2%)		
Positive	71(33.0%)	32(27.4%)	39(39.8%)		
**Ki-67**					
≤14%	51(23.7%)	27(23.1%)	24(24.5%)		
>14%	164(76.3%)	90(76.9%)	74(75.5%)		
**P53**				1.718	0.190
Negative	119(55.3%)	60(51.3%)	59(60.2%)		
Positive	96(44.7%)	57(48.7%)	39(39.8%)		
**Chemotherapy regimen**				4.478	0.107
Antracycline + Taxane	179(83.2%)	101(86.3%)	78(79.6%)		
Chemotherapy + Trastuzumab	24(11.2%)	13(11.1%)	11(11.2%)		
Other regimens	12(5.6%)	3(2.6%)	9(9.2%)		
**Chemotherapy Cycle**				0.007	0.935
<6	84(39.1%)	46(39.3%)	38(38.8%)		
≥6	131(60.9%)	71(60.7%)	60(61.2%)		
**Surgery**				0.318	0.573
Mastectomy	181(84.2%)	100(85.5%)	81(82.7%)		
Conservative surgery	34(15.8%)	17(14.5%)	17(17.3%)		
**pCR**				8.860	**0.003**
No	164(76.3%)	80(68.4%)	84(85.7%)		
Yes	51(23.7%)	37(31.6%)	14(14.3%)		

Bold values indicate that they are statistically significant at P<0.05

### 3.4 Relationship between PIV and HR subgroups

Based on the above results, HR status and PIV were independent predictors for pCR in young breast cancer patients treated with NACT. Subgroup studies were conducted to examine the link between PIV and HR status in more detail. In the HR positive group (n = 147), the pCR rate of the low PIV group was 19.0% (16 cases), and that of the high PIV group was 9.5% (6 cases). There was no significant difference in the probability of pCR between different PIV subgroups (χ^2 ^= 2.566, *P* = 0.109). In the HR negative group (n = 68), the pCR rate of the low PIV subgroup was 63.6% (21 cases), and that of the high PIV subgroup was 22.9% (8 cases). The probability of pCR was significantly different between different PIV subgroups (χ^2 ^= 11.548, *P* = 0.001) (**Table 5**). Our results implied that the young breast cancer patients with HR negative status and low PIV level were easier to achieve pCR.

**Table 5:** Relationship between PIV and HR subgroups.

Variable	HR- (n=68)				HR+ (n=147)			
	Non-pCR	pCR	χ^2^	*P* value	Non-pCR	pCR	χ^2^	*P* value
PIV ≤ 243.19	12	21(63.6%)	11.548	**0.001**	68	16(19.0%)	2.566	0.109
PIV>243.19	27	8(22.9%)			57	6(9.5%)		

### 3.5 Survival analysis

There was a 3- to 140-month follow-up period, and the mean survival time from the start of follow-up for 215 young breast cancer patients was 75.8 months. High clinical T stage (*P* = 0.011), cTNM staging level III (*P* = 0.028), high MLR (*P* = 0.023), and high PIV (*P* = 0.005) were all associated with a higher risk of death in univariate analysis. Nevertheless, cox regression models revealed only PIV as independently correlated with OS in multivariable analysis, and the prolonged OS time was shown in the low PIV group (OR = 2.523, 95% CI 1.005-6.334, *P* value = 0.049) (**Table 6**).

**Table 6:** Univariate and multivariate analysis of clinicopathological characteristics and IIBs in relation to OS.

Variable	Univariate analysis HR(95% CI)	*P* value	Multivariate analysis HR(95% CI)	*P* value
Age				
≤35	Ref.			
>35	0.940(0.435-2.032)	0.874		
BMI				
≤24	Ref.			
>24	1.154(0.524-2.544)	0.722		
cT stage				
T1+T2	Ref.		Ref.	
T3+T4	2.788(1.265-6.145)	**0.011**	2.137(0.902-5.063)	0.085
cN stage				
N0	Ref.			
N+	4.794(0.650-35.384)	0.124		
cTNM stage				
I-II	Ref.		Ref.	
III	2.637(1.109-6.274)	**0.028**	1.931(0.749-4.973)	0.173
HR				
Negative	Ref.			
Positive	0.706(0.320-1.555)	0.387		
HER-2				
Negative	Ref.			
Positive	1.492(0.685-3.249)	0.313		
Ki-67				
≤14%	Ref.			
>14%	2.524(0.758-8.408)	0.131		
P53				
Negative	Ref.			
Positive	0.900(0.413-1.960)	0.791		
Chemotherapy regimen				
Antracycline + Taxane	Ref.			
Chemotherapy + Trastuzumab	0.299(0.040-2.209)	0.237		
Other regimens	0.634(0.086-4.686)	0.655		
Chemotherapy Cycle				
<6	Ref.			
≥6	1.764(0.742-4.197)	0.199		
Surgery				
Mastectomy	Ref.			
Conservative surgery	0.435(0.103-1.842)	0.258		
NLR				
≤1.55	Ref.			
>1.55	3.761(0.889-15.913)	0.072		
PLR				
≤130.66	Ref.			
>130.66	1.471(0.667-3.241)	0.339		
MLR				
≤0.24	Ref.		Ref.	
>0.24	2.502(1.135-5.515)	**0.023**	1.891(0.815-4.388)	0.138
PIV				
≤243.19	Ref.		Ref.	
>243.19	3.466(1.457-8.247)	**0.005**	2.523(1.005-6.334)	**0.049**

Bold values indicate that they are statistically significant at P<0.05

**Figure 1:**
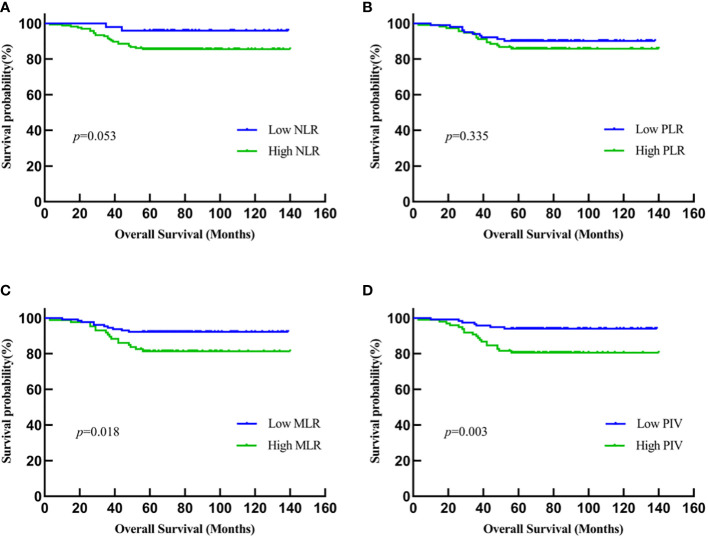
Kaplan-Meier analysis of the relationship between IIBs and OS. **(A) **NLR; **(B) **PLR; **(C) **MLR; **(D) **PIV.

Kaplan–Meier methodology and log-rank test showed that the OS time in young breast cancer patients with low MLR and low PIV before NACT was significantly longer than those with high MLR and high PIV (MLR: *P* = 0.018; PIV: *P* = 0.003). This trend persists in both short-term and long-term prognosis, suggesting the prognostic impact of MLR and PIV in young breast cancer patients treated with NACT. By contrast, the other two IIBs, NLR and PLR, were not significantly related to OS (NLR: *P* = 0.053; PLR: *P* = 0.335) (**Figure 1**). As the follow-up period increased, the patients with low NLR and low PLR may be significantly longer than those with high NLR and high PLR.

## 4 Discussion

To our knowledge, this is the first study demonstrating PIV’s prognostic significance in young breast cancer patients treated with NACT. This study first showed that PIV was an independent predictor of pCR and OS, whereas the patient’s outcome was not substantially correlated with the NLR, PLR, and MLR. Since the majority of our patients had cT2 stage, node-positive, and cTNM III stage breast cancer, our cohort was typical of those young breast cancer patients undergoing NACT. One notable advantage of our series lay in its long clinical follow-up, spanning a median period exceeding six years, which was far longer than that of most comparable studies. It enabled a mature and pertinent evaluation of survival outcome.

This increased lifespan is linked to a progressive deterioration in immunological function and chronic inflammation (24). Immune aging is primarily manifested by these modifications in T- and B-cell composition and function (25). What’s more, the defective immune response is mainly characterized by thymic involution (25), bone marrow degeneration (26), and aging lymph nodes (27). Compared to those who are older, youngsters experience fewer innate and adaptive immune response modification changes by aging (28). However, there are relatively few studies concerning immune and inflammation, solely in young cancer patients. Our research revealed that the low level of PIV group was more easily to achieve pCR and had longer OS in young patients with breast cancer. Inflammation has been widely recognized for becoming detrimental at some point in the elderly (28), and low levels of inflammation help the body neutralize foreign detrimental agents in healthy youngsters (17, 19). However, high inflammation level was intricately associated with poor prognosis in our study. It seems that when the tumor has not yet developed, the immune-inflammatory system assumes the role of a tumor suppressor, while in the presence of a fully formed tumor, the immune-inflammatory system acts as a tumor promoter in young breast cancer patients. It reflects that the immune-inflammatory system may affect a patient’s prognosis through a local immune response and plays different roles in the different periods of tumor formation. In order to better understand its part in cancer formation and progression, more human studies are necessary in the future.

Various studies have demonstrated that the immune-inflammatory system plays a pivotal part in the proliferation, invasion, and metastasis of tumors (29–31). IIBs from peripheral venous blood can reflect the status of the whole immune-inflammatory system. Neutrophils participate in tumor invasion and metastasis by releasing inflammation intermediates, for instance, matrix metalloproteinase-9, neutrophil elastase, and interleukin-8 (32–34). Monocytes can alter the tumor microenvironment by inducing angiogenesis, immune tolerance, and cancer cell dissemination (35). Platelets promote the growth and metastasis of cancer through platelet-derived growth factors (36). In contrast, lymphocytes are essential to the anti-tumor immune response that prevents tumor growth and metastasis (37). PIV is based on the four types mentioned above of inflammatory cells; hence, it can sufficiently assess the state between host immunity and inflammatory status. Many academic works have discussed the prognostic value of PIV in various cancers. Feng et al. confirmed that PIV was associated with the tumor stage and prognosis in esophageal squamous cell carcinoma patients undergoing radical resection (38). Zhai et al. found that PIV can predict pCR of patients with non-small-cell lung cancer who received neoadjuvant immunochemotherapy (39). A meta-analysis demonstrated that colorectal tumor patients in the high pretreatment PIV group had poor OS and progression-free survival (40). Several researches supported the predictive role of PIV in breast cancer (15, 41). Among a population of patients with advanced triple-negative breast cancer undergoing platinum-based chemotherapy, Provenzano et al. found that both the baseline and initial on-treatment PIV correlate with OS (41). Our study also demonstrated that PIV was significantly related to pCR and OS in breast cancer patients undergoing NACT. More importantly, our population was entirely different from theirs since we only included young patients (≤ 40 years old).

Apart from PIV, our study’s findings partially conflict with the already available literature, involving NLR, PLR, and MLR. An updated meta-analysis of 17079 breast cancer patients suggested that NLR and PLR were connected to a high risk of recurrence and poor OS, particularly in triple-negative breast cancer (12). Nevertheless, Suppan et al. could not show that NLR had predictive or prognostic significance in the group of patients with early-stage breast cancer receiving NACT (42). Similarly, Losada et al. found that PLR ≥150 was not associated with 5-year OS in elderly breast cancer patients (43). In this study, the high NLR and high PLR groups have lower pCR rates upon univariate analysis. Upon multivariable analysis, we did not find a significant relationship between NLR or PLR and pCR. We hypothesized that young women have a potential role in modulating systemic inflammation and can overcome the adverse prognostic effects of high baseline NLR and PLR. Another possible reason was that part of the population used the herceptin and pertuzumab in this study. Meng et al. reported an association between MLR, chemotherapy response, and prognosis (44). However, we only found that a high MLR may have an adverse effect on OS by Kaplan–Meier methodology and log-rank test. In our study, PIV was not a predictive indicator for pCR. All these conflicting results may emanate from differences in the study cohorts and selection bias.

In this study, PIV was negatively correlated with pCR and lacked statistical significance with other clinicopathologic features. Patients with low PIV were more likely to get pCR than those with high PIV, suggesting that PIV has a potential predictive value for chemotherapeutic response. Since patients in the high PIV group may be in a tumor-immune-inflammatory state, it is difficult to correlate this state with separate clinicopathologic indicators.

Younger women with BC are a relatively small yet clinically extremely distinct subgroup who are unlikely to have a contraindication to undergoing NACT. About one-fifth of young breast cancer patients received NACT in China over the past two decades (23). There were two studies investigated young breast cancer patients undergoing NACT with anthracyclines/taxanes-based NACT regimens with or without targeted anti-HER-2 agents, pCR rate was 20.3% (7) and 20.9% (45), respectively, which were similar to our study data (23.7%). Li et al. found that molecular subtype and Ki-67 index were independently associated with pCR in young patients, which was consistent with our results (7). In addition to this, we further discovered that the pCR rate was significantly higher in the population of HR negative status and low PIV level. Wang et al. detected that the 5-year survival was 91.6% in young patients with breast cancer under 35 years old (23). In our study, the OS rate over five years was 87.9%. The reasons for this discrepancy in survival data may be disparate age ranges and pathological characteristics of the population.

We acknowledge that there are some limitations to this study. Firstly, it is a retrospective study with a monocentric design. More individuals should be included in future multicenter research. Secondly, systemic therapy approaches have evolved over this extensive study period, for instance, the large-scale use of targeted drugs for HER-2 positive breast cancer and neoadjuvant immunotherapy for triple-negative breast cancer (46). Thirdly, above one third of patients were in the high body mass index (BMI) group, given the potential fat mass effect on the immune system (47). Lastly, different cut-off points of IIBs may affect the prognostic value in different research.

## 5 Conclusion

Our research thoroughly explores the prognostic value of IIBs and finds that PIV is associated with pCR and OS in young breast cancer patients undergoing NACT. Besides, this research suggests that HR status, HER-2 status, and Ki-67 index are all independent predictive factors for pCR in young breast cancer patients, especially the patients with HR negative status and low PIV level, are easier to achieve pCR. In contrast to the NLR and PLR, which are not substantially associated with the young patient’s prognosis, MLR is a good predictor of 5-year OS. Additional research is required to fully comprehend the different roles of the immune-inflammatory system between healthy young people and young cancer patients. Young patients with breast cancer have a worse prognosis; thus, we need to discover novel biomarkers and attempt to combine new biomarkers with PIV to improve clinical outcomes.

## Data availability statement

The original contributions presented in the study are included in the article/supplementary material. Further inquiries can be directed to the corresponding author.

## Ethics statement

The studies involving humans were approved by Harbin Medical University Cancer Hospital. The studies were conducted in accordance with the local legislation and institutional requirements. The participants provided their written informed consent to participate in this study. Written informed consent was obtained from the individual(s) for the publication of any potentially identifiable images or data included in this article.

## Author contributions

FL: Conceptualization, Data curation, Investigation, Methodology, Software, Visualization, Writing – original draft, Writing – review & editing. YW: Conceptualization, Data curation, Formal analysis, Methodology, Project administration, Writing – review & editing. HD: Conceptualization, Formal analysis, Investigation, Software, Supervision, Validation, Writing – review & editing. XC: Data curation, Methodology, Supervision, Writing – review & editing. JW: Writing – review & editing. MX: Conceptualization, Formal analysis, Funding acquisition, Project administration, Resources, Supervision, Validation, Writing – review & editing.
